# Child Development and Early Interaction: PERL Research Protocol, a Preventive Home Visiting Program, Randomized Controlled Trial in France

**DOI:** 10.3389/fpsyt.2021.641468

**Published:** 2021-06-30

**Authors:** Sophie Buchheit, Bernard Kabuth, Marie-Christine Colombo, Fabienne Ligier

**Affiliations:** ^1^Pôle Universitaire de Psychiatrie de l'Enfant et de l'Adolescent, Centre Psychothérapique de Nancy, Laxou, France; ^2^Université de Lorraine, Laboratoire EA 4432 InterPsy (PRISME), Nancy, France; ^3^Service Départemental de PMI, Direction des Solidarités, Conseil Départemental de Meurthe-et-Moselle, Nancy, France

**Keywords:** prevention, home visiting, early interaction, development, early childhood, randomized controlled trial

## Abstract

**Background:** Early childhood is a key period for reducing the social inequalities that affect health. Some parenting support and home visitation programs have proven to be effective in assisting parents during this period. France's *Protection Maternelle et Infantile* (maternal and child welfare) services (PMIs) are at the heart of this primary prevention and may adapt their intervention to improve parenting support. In this manuscript, we describe the protocol of the PERL study, an intervention based on a home visiting program.

**Method and design:** The PERL study is a single-center, randomized, controlled interventional trial. The aim was to assess the impact of a preventive home visiting program on the development of young children and parent-baby interaction. Visits were made by PMI nurses to 64 randomly recruited families from the general population. All families who had a baby born after 37 weeks of pregnancy between September 2018 and December 2019, and who resided in the trial area were eligible. Participants were randomly allocated to the intervention group or the control group. The PMI nurses made 12 home visits in the first year, 6 in the second year, and 4 in the third and fourth years of the child's life. Primary and secondary outcomes were measured when the child was 4 and 24 months old. These measurements recorded (i) the child's developmental milestones, in particular, language and socio emotional skills, (ii) early interaction, maternal sensitivity, and attachment patterns, (iii) maternal psychopathology including depression.

**Discussion:** This study aims to assess the impact of home visits, made by specifically-trained and supervised nurses, on the child's development and parent-child interactions. Such interventions are complementary to other preventive programs addressing the impact of social inequalities on perinatal health. Placing nurses' professional skills at the center of this project may prove an effective and cost-saving intervention compared to existing programs. The study proposes a prevention model that is in keeping with the principle of reducing social inequalities in health by providing support from the earliest age through public service.

**Clinical Trial Registration:** The clinical trial number is NCT03506971, registered on April 24, 2018.

## Background

### Early Childhood

Public health policies encourage primary prevention. Early childhood is a key period for reducing health social inequalities, and a priority in French health policy (1). Parenting support is promoted because it is recognized as a potentially valuable action (2–7).

In the first 3 years of life, a child goes through a period of dynamic growth (communication, interaction, psychomotor, and emotional development, etc.), which is considered to be the most important developmental stage in their life (8). It has been proven that the quality of a child's development during this early period is correlated with social interaction and mental health throughout life (9).

### Early Intervention

Literature shows the positive effects of early intervention in infant mental health prevention (10, 11). Early intervention is defined as the treatment of children under the age of 2 years and their parents in a therapeutic setting, to foster early attachment bonds (12).

Focusing on prevention, interventions to prevent and promote infant mental health issues are mostly intended for families deemed to be “at risk” (13–16). They can focus on the parent, child, and parent-child interaction or family, social services, and community support. These interventions have a positive effect on child development, parenting skills, parent self-esteem, parent-child attachment, bonding, and access to support and welfare services.

### Home Visits

Early prevention programs that include Home Visits (HV) incorporate the family environment into care, i.e., an environment that is familiar and often more reassuring for families (17). These programs are expected to reduce the impact of social stress factors on families, and promote parenting skills, especially in vulnerable families. Home-based parent-baby intervention generates changes, for example, parents are often more independent, with improved parenting skills, mothers are less depressed, attachment is more secure, and there are fewer behavioral disorders (16).

HV has been proven to be effective, with positive results in terms of the child and mother's mental health, early bonding, and more globally, on child development (18–20). The results of the STEEP (Steps Toward Effective, Enjoyable Parenting) program show that, at 24 months, parents who benefited from HVs had a better understanding of their baby's crying patterns, feel more positive about themselves, are less depressed, focus more keenly on pleasurable activities, and shared well-being involving the baby (21).

Moreover, a combination of weekly HVs with professional supervision has a substantial impact, including greater maternal empathy, reduction in avoidance or resistant behavior in young children, and the positive introduction of parent-child attachment bonds (22).

### HV and Prevention in France

The French government recently set up the First 1,000 days of life commission (23), highlighting the importance of this stage in a baby's development and the support needed by families during this crucial period.

In France, studies have tested the effectiveness of HVs in the perinatal period (24–26) (Table 1). We built the PERL study protocol drawing upon the strengths of these studies with the specific aim of proposing a protocol that can be blended into current practices (section Methods and Analysis).

**Table 1:** French research on HVs.

	**CAPEDP (26)**	**INTERREG (27–29)**	**PANJO (30, 31)**
Characteristics	HV	HV	HV
Objective	Assess the impact of HVs carried out by attachment-trained psychologists on postnatal depression, the quality of the home environment and the psychological disorders	Evaluate the impact of HV carried out by a psychologist on language delays and child development	Adapt and experiment in PMI services a promotion system health, attachment support and evaluate the parent-child attachment
Population concerned	young mothers (<26 years) with vulnerability criteria	General population across 3 counties of the Lunevillois area	Primiparous and isolated mothers
Duration of study	24 months	24 months	6 months (up to 12 months if necessary)
Number of HVs	44	23	6 (until 12)
Professional	Psychologist	Psychologist	PMI nurses
Characteristics of the setting	- Training on the attachment theory, community psychology, developmental guidance, use of home video - Weekly individual supervision	−2 supervisions per month	- Training at the attachment theory - Supervision (frequency not specified)
Characteristics of the content of the HVs	- Observation - Discussion with parents and child Use of specific tools: video, playing cards to enable verbalization - Intervention mainly focused on the quality of the parent-child attachment and process related to attachment	- Observation - Interaction with parents and parent-child interaction through play - The content was mainly non-directive and not “interventional” - Based on psychoanalytic theoretical basis	- Observation - Discussion with parents and child - Use of attachment intervention guide and specific tools: video, playing cards to enable verbalization - Intervention mainly focused on the quality of the parent-child attachment and process related to attachment
Results	Does not show any significant difference in postpartum depression at 3 months vulnerable mothers - Highlights a better use of the care network (mainly PMI) - Highlights a better buy in from families when in their social environment	- Reduction in language delay - Significantly better psychomotor development at 2 years. - Significantly reduction in the number of children referred for psychological and/or speech therapy in the participant cohort at 4 years old	- The quantitative results of this research have not yet been published - The program was seen by the professionals of PMI as beneficial for their practice, even if the program supported interventions of the PMI among families and promoted a better understanding of the missions of PMI

### What Is the PMI Service?

The PMI is the maternal and child welfare service of France. It is a free public service for all French families, run by early childhood professionals. One of the main tasks of the PMI is to make primary prevention universal through HVs. PMI nurse interventions are based on guidance, providing advice on nutrition, development, the sleep/wake cycle of the baby, and so forth.

A previous national study has highlighted that PMI HVs are rarely part of a structured program (so typically lack stated objectives, guidelines, framework, training, etc.) (32, 33).

### The Content of HVs in Studies

HVs are centered on child development, the strengthening of parenting skills, the quality of early parent-child interactions (34), and the impact of the health professional on these aspects, according to the model of Fraiberg (12).

One of the main support criteria is based on the quality of the relationship between the support-giver and the parent (35): “most situations are first and foremost in need of a human/humanist touch, and this is very often all it requires” (36). Through regular HVs, the professional bolsters the security of the family bond by offering a constant and reassuring attachment, becoming an attachment figure for the parent (37). A PMI nurse may be used as a “resilience mentor” (38) by creating a temporary safe base (39, 40). This allows the parents to experiment, both mentally and physically, with a secure relationship which they can then transfer into the relationship with their child.

Several English-language studies (41, 42) have found that an adult who experienced an adverse childhood event needs to forge a rewarding relationship with the therapist. These parents are present in the general population. The professional's confidence in the ability of both parent and child can boost not only their confidence in themselves but also in the support available to them (43).

According to the literature, three key HV concepts are important:

(1) Parenting support: the professional listens actively and attentively, facilitating the emergence of imaginary and narcissistic representations relating to the baby. This parental support would enable a secure attachment to be formed among the children. Five components make up the relationship with the caregiver, and increase parents' receptiveness toward their baby (12, 14, 44, 45):

- Emotional support for parents;
- Assistance with resources and requirements;
- Developmental guidance for encouraging and strengthening the quality of attachment between parents and baby, helping parents to understand the “language” of their child and devising new educational methods;
- Infant-parent psychotherapy: the exploration of the thoughts and feelings which are awakened in the baby's presence;
- Use of the “echo function” (46) to speak for the children who cannot speak and where the parents are too overwhelmed by their emotions.

It is important to strengthen parenting skills (47). It is important to “take care of the person who takes care of others” (48). Listening to the difficulties encountered by the parents, and their need for reassurance, allows the parents to rekindle their ability to protect and support the baby (12, 48, 49).

(2) Observing the baby: Observation is considered an effective therapeutic action (50). Observing babies and their progress enables parents to identify with the “observing function” and so improve their child's development. According to Fraiberg (12), we give attentive and empathic responses to the parents, which can then be applied by the parents to the relationship with their child, paying particular attention to the emotions and behavior associated with past experiences. Active, attentive watching during observation can provide reassurance and strengthen the parent-child relationship (51, 52).

(3) Encouraging parent-baby interaction: as attachment theory has shown (39), from birth babies are social beings for whom interactive exchanges with a caregiver represent a primary need. Health professionals must therefore be interested in gradually building attachment bonds, and invite the parents to form a relationship with the baby through care, play, or active observation.

Play is a fundamental mediator in this interaction, allowing exchanges through different methods (visual, vocal, physical) and engaging each of the partners in a relationship that brings mutual pleasure. It represents a real “emotional bridge between parent and child,” the parent helping the child to develop through play (53, 54). Play may also be therapeutic in its own right (55, 56). Some therapeutic strategies focus almost exclusively on parent/child play to facilitate development and introduce a secure parent/child relationship (57).

With PERL, we propose follow-up interactions that encourage the child's development, strengthen the parents' ability to cope with their little one, and highlight the development and importance of early parent-child relationships (12, 44).

In the PERL study, HVs are included in a structured program, with guidelines, working methods, and professional training.

### Objectives of the PERL Study

The main objectives are to assess the effects of regular preventive HVs performed by PMI nurses on the prevalence of developmental and language delays in children, by comparing intervention and control groups.

The secondary objective is to longitudinally analyze the impact of the intervention for each family over the child's fourth to 24th months. The impact will be studied through the evolution of the child's development, looking at therapeutic alliance, parental feelings about the intervention and maternal mental health, quality of mother-child interactions, and experience of parenting.

PERL is an intervention system because a professional accompanies the family with tailored support. It is also a prevention system because this intervention begins before any disorder or diagnosis of delay. The goal in terms of mental health protection is to reduce the incidence of disorder by eliminating or reducing risk factors, and by intervening before symptoms develop. We propose with PERL a primary prevention program through intervention.

### Outline of the Trial and Hypotheses

The PERL action-research study is a single-center, randomized, controlled, interventional trial over a 4 year period that compares a group receiving regular preventive monitoring at home with a group that does not receive this intervention.

We expect a positive effect from the HVs performed by specifically-trained and supervised PMI nurses on:

- Children's development: we expect to observe for the intervention group at 24 months less developmental delay, less relational withdrawal, and fewer social and emotional difficulties. We also expect participants in the intervention group to have at 4 years a lower prevalence of language delay compared to the control group.
- Parent-child interaction: we expect to find in the intervention group at 24 months greater reciprocity and continuity in interaction, better emotional relationship, and more maternal behavior, which facilitates exploration of the environment.
- Experience of parenting: we expect in the intervention group at 24 months to observe more descriptors and positive markers in the mother's speech
- Maternal mental health: we expect to find in the intervention group at 24 months less depression and fewer psychiatric symptoms.
- Attachment: we expect to find in the intervention group at 24 months more secure attachment.
- Maternal sensitivity: we expect to observe in the intervention group at 24 months a more “sensitive” mother profile in comparison with the control group.

## Methods and Analysis

In our opinion, the research presented in Table 1 has several limitations in terms of:

- the selection of a specific population (26, 35, 36);
- reliance on professionals who are not usually involved in this preventive care (26, 32–34);
- having a follow-up of ≤ 24 months (26, 32–36);
- being based only on very specific theories (26, 32–36).

For PERL, we drew on the strengths of previous studies (cited in Table 1), bypassing the limits and choosing to offer intervention:

- in the general population;
- by relying on existing practices;
- using an eclectic theoretical model;
- over a longer period, until the child reaches 4 years old.

### Sample

#### Context of the Lunevillois Area

The study was located in a rural, semi-rural, and urban area of eastern France called “Lorraine,” and was named the PERL study: *Petite Enfance, Recherche-action en Lorraine* (*Early childhood, action-research in the Lorraine area*). The context of the Lunevillois area is described in Table 2.

**Table 2:** Context of the Lunevillois area.

**On 31.12.2018**	**Lunévillois**	**France**
Employment rate 15–64	62.8%	63.9%
Unemployed persons 15–64	13.8%	13.6%
Inactive people 15–64	27.1%	26%
Persons who benefit from an income of Active Solidarity (RSA) (for 1,000 persons 15–64 years)	42.4%	40.6%
Density of general practitioners (for 10,000 persons)	10.5	9
Number of PMI's consultations	73%	83% (in Lorraine)
Children with social or justice support (minor placed, educator,…) for 1,000 children under 20 years	32%	26.7% (in Lorraine)
School enrolment 2–5 years	73.5%	73.7%

*Lunévillois area: 1,576 km^2^, 178 towns, 67 inhabitants per km^2^*.

#### Determination of Sample Size

A calculation of sample size was carried out with one case per one control, a power of 80%, an alpha risk of 5%, a global developmental delay (obtained with the BLR test) prevalence rate of 50% in the controls and 25% in the children on the program (based on INTERREG results). The result obtained is a sample of 58 people in the intervention group and 58 in the control group. Taking into account the potential loss of 10% of subjects over the course of this ongoing research, 64 subjects were originally recruited for the intervention group and 64 subjects in the control group.

#### Sample Characteristics

- Eligibility criteria

There were two eligibility criteria: (i) the child was to have been born between September 2018 and December 2019 in the general population; and (ii) all families had their main residence in the *Pays Lunévillois*, in Lorraine, France.

- Exclusion criteria

There were three exclusion criteria: (i) a child born prematurely, before 37 weeks of pregnancy; (ii) families that did not speak French; (iii) non-settled traveler families, temporary refugees, and families with no fixed abode.

Participants were not prohibited from taking part in other research projects during this trial.

**Figure 1:**
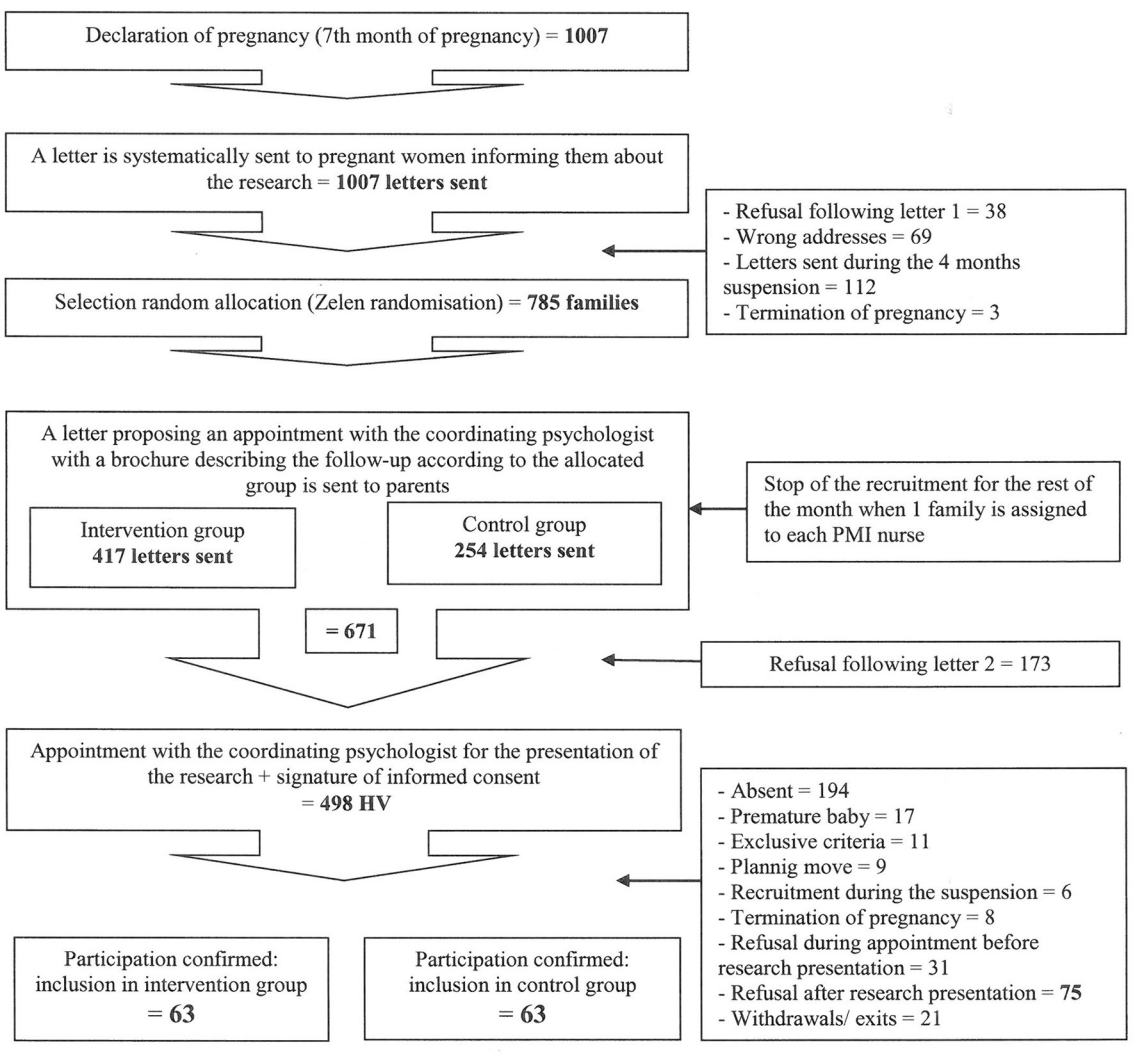
Recruiting method of the PERL study.

- Randomization (Figure 1)
1) Every month, the PMI service sent the coordinating psychologist (SB) a list of all pregnancies reported in the population residing in the recruitment area, in the seventh month of pregnancy (on average 60 births per month).
2) SB then sent a letter to all of the families on the list, informing them that research was being conducted and that they were likely to be contacted and invited to participate. This letter informed the families that they had 2 weeks to refuse participation.
3) Two weeks later, SB performed the randomization process (Zelen randomization with a double consent for the 2 groups) based on the updated list of reported pregnancies post-refusal and returns for wrong address after the first letter had been sent. The families were thus randomly assigned to either the “intervention” or the “control” group.
4) SB then sent a second letter informing families that they have been randomly selected to take part in the PERL study and forwarded a brochure describing the terms of participation (purpose of the study, how to participate, a presentation of the relevant stakeholders) with a suggested date for an HV (the start of the 8th month of pregnancy).
5) If no refusal was received from the families, SB performed the HV to outline the trial to them, and if they agree to take part in the study they were asked to sign consent forms.

This recruitment method was highly formalized to meet the needs of research. Families were randomized in accordance with Zelen randomization before consent was requested to prevent the families' preference for one or another group and to avoid disappointment at not being in the intervention bracket. However, all parents may benefit from the PERL study: control group parents still had two assessments of their child's development, which could be reassuring. As double-blind evaluation cannot be performed in the PERL study, to circumvent this limitation, analysis will be performed by a professional who does not know the attribution group.

#### Rate of Inclusion

The PMI nurses in the area have taken on the PERL system in addition to their normal workload. As a result, we have had to adapt the number of families followed by each nurse to match the resources allocated by the PMI service. The PMI nurses were able to take on up to four families each, with 16 PMI nurses taking part in total. The control group was recruited with the same number of participants each month in order to avoid any bias (seasonality, etc.) and so that SB could perform the evaluation HVs within the allotted time. When the expected number of families per month (1 per PMI nurse) was reached, recruitment then stopped for that month and resumed the following month. In practice we had to limit the number of study participants to 128 families to enable coordination and feasibility in the field.

The number of PMI nurses participating in the project changed over the months (from September 2018 to September 2019). There were 6 PMI nurses over the first 3 months, 9 the following 7 months, then 10 professionals from May 2019, and at least 16 from September 2019.

The maximum number of subjects recruited has therefore changed according to the number of professionals involved in the research, 16 professionals being the necessary number to recruit the entire population. Furthermore, for reasons outside the study, recruitment was interrupted for 4 months (January 2019 to April 2019).

If we had been able to involve the desired number of professionals at the start of the research, we would have reached our recruitment goals within 5 months.

#### Families Support

##### PERL Support Intervention Group

**Figure 2:**
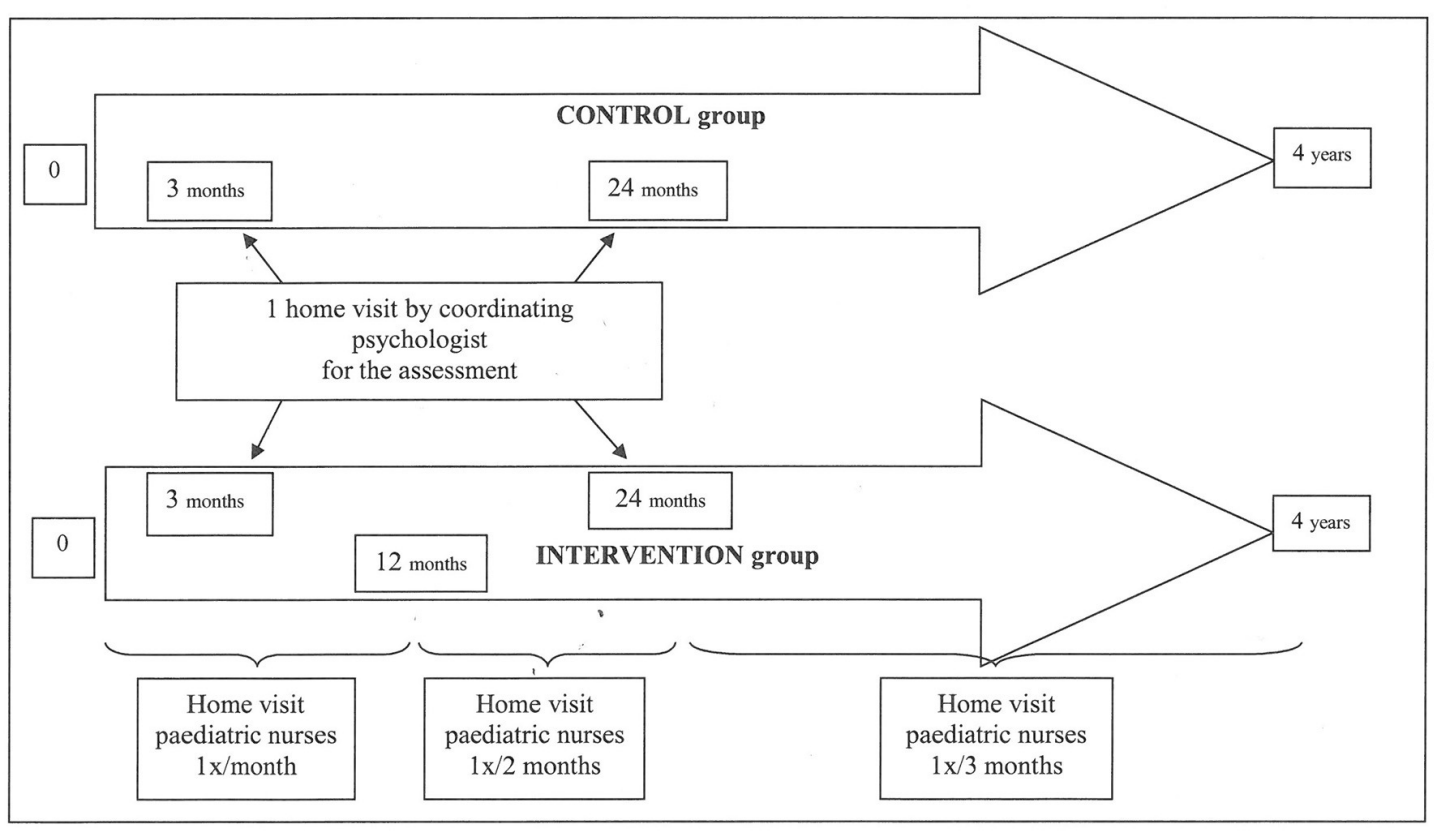
Study design of the PERL study.

Following the birth, families in the PERL “intervention” group receive HVs from a PMI nurse divided into three stages: (i) active observation of the baby's development and progress, (ii) playing with the baby, and (iii) listening and talking to the parents (parenting support). The families support is described in Figure 2.

These HVs take place at predefined intervals (1 HV every month from birth up to the child's 1st birthday, 1 HV every 2 months from 1 to 2 years of age, and 1 HV every 3 months from 2 to 4 years of age) as shown in Figure 2, totaling 26 HVs over 4 years.

The work of PMI nurses is focused on unconditional kindness and availability, enhancing parenting skills, non-directive action, responsiveness to parents' needs and questions, parental contact and support, having a positive approach to situations, and the building of a secure parent/pediatric nurse relationship.

*Particular Case*. Intervention concerns all families residing in the area targeted by the study (universally), irrespective of the make-up of the family (nuclear or single-parent families). Support and assessment will be provided under the same conditions for a single-parent.

At the inclusion stage, the smallest child of the family is included. If during the study a birth should occur in the family, the study will continue without the inclusion of the newborn. Only 1 child in the family is assessed, from birth (4 months) to 4 years.

If other children are present (physically or in conversation), the PMI nurse can meet them: the goal is to respond to parents' requests and to support them throughout their parenthood.

Should the father wish to be present during the HV, they are welcome to do so. Other parents are able to attend HVs should the mother be unavailable; however, the evaluation focuses only on the mother to ensure comparability of certain gender-sensitive tests.

##### Control Group

There is no specific intervention for families in this group, with the exception of the recruitment HV carried out by SB and the two evaluations planned in the study.

While a family in the control group can benefit from monitoring systems should they need to do so (midwife, conventional PMI consultations, maternity hospital, psychological medical center, social worker, and pediatrician, etc.), this is not specifically proposed through the PERL study.

##### Evaluation in Both Groups

To check the initial homogeneity of the groups, we proposed an early comparison of the groups with 10 families from each group, randomly selected by a double-blind process. An independent psychologist assessed the baby's development during the first 30 days after birth.

Two HVs are carried out by SB (and the family's dedicated PMI nurse in the “intervention” group), with the parents, taking place when the child is 4 and 24 months old. The purpose is to assess three things: the child's development, parenting, and parent-child interaction. The assessment tools are described in Table 3.

**Table 3:** Outcome criteria and assessment schedule.

**Instrument**	**Concept measured**	**Validation**	**Outcome assessment place of evaluation**	**Structure of instrument; scoring**	**Time of measurement**				**Group**	
					**0**	**4**	**24**	**4**	**I**	**T**
					**Mths after birth years**					
Neonatal Brazelton Assessment Scale (NBAS)	Neonatal behavior (0–1 month)	Brazelton e (58) (3° ed)	Psychologist at the maternity hospital/home visit	The assessment concerns: autonomic system motor tone organization state organization and regulation early interaction	X				X	X
Brunet-Lézine Revised Developmental test	Child development (2–30 months)	Brunet and Lézine, 1965 (59)	Psychologist during home visits	Developmental age (developmental quotient)4 dimensions: Language Social relationships Motor gross Motor fine 30 item test scored partly		X	X		X	X
Alarm Baby Distress scale (ADBB)	Sustained withdrawal behavior (2–24 months)	Original scale in FrenchGuedeney and Fermanian (60) in France	Psychologist, after home visits, with video	8 items, 5 point Likert scale (0–4), range 0–32 Withdrawal behavior if ≥ 5		X	X		X	X
Symptom Check-list (SCL-90)	Mother's psychological disorders	Derogatis (61) Translation	Mother during home visits	90 items, 5 point-Likert scales (0–4) X X X10 subscales: Somatization (0–48) Obsessive-Compulsive (0–40) Interpersonal Sensitivity (0–36) Depression (0–52) Anxiety (0–40) Hostility (0–24) Phobic Anxiety (0–32) Paranoid Ideation (0–24) Psychoticism (0–40) Other symptoms (0–28) Higher scores indicate greater Clinical impairment.		X	X		X	X
Working Alliance Inventory (WAI)	Working alliance between the mother and the pediatric nurse	Horvath and Greenberg (62) French validation Guedeney et al. (63)	Mother and pediatric nurse after home visits	12 items, Likert scale (1–7) Range 12–84 Higher scores indicates better working alliance		X	X		X	
Attachment Q-Sort (AQS)	Child's attachment (10 months−3 years)	Waters and Deane (64) French translation made and validated by a panel of infant mental health experts (65)	Assessment team (psychologist and nurse) after home visits	Two rates assess each situation during a home visit of 2 h90 itemsCorrelation with typical secure pattern: Insecure attachment if <0.35 Secure attachment if ≥ 0.35			X		X	X
Edinburgh Postnatal Depression Scale (EPDS)	Self-Assessment of postpartum depression	Cox et al. (66) French validation: Guedeney and Fermanian (67)	Mother during home visits	10 items, 4 point-Likert scales (0–3) Range 0–30 Higher scores indicate higher levels of depressive symptoms.		X			X	X
Bobigny's early interactions grid	Mother-infant interactions (0–4 years)	Devised by experts in infant mental health and specifically perinatal consultations, 1989 (68)	Psychologist after home visits	Qualitative assessment grid describing the quality of the interactions and the dominant mode of interaction.		X	X		X	X
Mini Q-SORT	Maternal sensitivity	Pederson and Moran (69) Suited to analysis of short video sequences Tarabulsy and et al. (70)	Psychologist after home visits (video)	Sorting of 25 cards describing maternal behavior according to a Q-sort procedure		X	X		X	X
Beck Depression Inventory (BDI)	Maternal depression	Validated by Beck et al. in 1961 (71) Version abridged by Beck et al. in 72 (72) Translated and validated in French by Delay and Pichot in 1963 and 1964 (73, 74) Abridged version validated by Reynolds and Gould et al. in 1981 (75)	Mother during home visits	13 items 4-point Likert scales Range 0–39 Higher scores indicate higher levels of depressive symptoms. 4: no depression; 4–7: mild depression; 8–15: moderate depression; 16+: severe depression			X		X	X
Family Affluence Scale (FAS) form	Socio-economic status of the families		Mother during home visits	4 questions to tick: yes/no and enter number		X	X		X	X
Brief Infant-Toddler Social and Emotional Assessment (BITSEA)	Early screening for delays in social and emotional competencies (1–3 years)	Validated by Briggs-Gowan and Carter and (76) Translated by Wendland and Saïs, 2010 and validated in French by Wendland et al. (77)	Mother during home visits	42-item questionnaire 3-point Likert scales (0–2) Provides indications on the social-emotional profile and competencies of children according to their parents.			X		X	X
Parenting representations interview (PRI)	To obtain parents' representations concerning their child, themselves as parents and their experience of the intervention	Questions taken from the R interview drawn up by Lebovici et al. (78)	Mother during home visits	Semi-structured interview 4 questions. The themes are: description of their child, themselves as parents, their reaction to their child's crying, and their experience of the support and home intervention as part of the research		X	X		X	X
Language delay screening test (ERTL 4)	To assess language delays at age 4 years	French scale validated in 1998 (79)	The pediatric nurse during a systematic examination at nursery school	5 tests (2 optional), scoring with 3 categories: red (indication of further testing); orange (active monitoring, re-evaluation necessary); green (no difficulty)				X	X	X

Assessment of any language delay is undertaken in all schools in France by the PMI service, during the second year of kindergarten (at 4 years old).

If disorder or delay is observed in the child or the parent (such as postpartum depression, depressive symptoms, development or language delay, conjugopathy, addiction, etc.) then parents in the control group who are encountered at 4- and 24-month evaluations are referred to specialist services. For the intervention group, the evaluation HV process is identical. For this group should a delay in the child or parental mental disorders be noted by the PMI nurse then the family is immediately referred to a specialist service while remaining in the PERL system. SB is also informed in order to note the orientations carried out within the framework of the mechanism.

#### Accompaniment for PMI Nurses

##### Supervision: Systemized and *ad-hoc*

The PERL study's PMI nurses receive monthly supervision from a child psychologist and SB.

PMI nurses can contact SB at any time to report any difficulties encountered with the families. Individual supervision is also offered on request.

In the PERL study these supervisions sustain the unconditional kindness and necessary framework, and so are essential in support of the families (80, 81).

##### Training

All PMI nurses received initial training on the fundamental principles of support relationship and the factors that facilitate or inhibit it; the fundamental components of perinatal support; the “echo function”; and the principles of communication, attachment, therapeutic alliance, and their variations. There is also ongoing education to ensure awareness of rating scales, analysis of videos based on interaction and the baby's sustained withdrawal behavior, theoretical contributions about play, attachment, postpartum depression, unconditional kindness, and the non-directive-approach, the place of advice and guidance in the HVs, etc.

##### Working and Exchange Days

The PMI nurses attend quarterly working and exchange days where they review their roles and undertake 1 h a month of continued training.

The theoretical model proposed in this research program is intended to be eclectic: the fundamental principle of therapy described by Rogers (82), systemic and family theory (56, 83–85), free drivability (86), attachment theory (39, 87, 88), mother-baby therapies developed by Fraiberg (12) and Dolto (89, 90), etc., to offer PMI nurses a range of practices and interventions for use with the families. This model will be enhanced over the course of the meetings between the PMI nurses and SB, in line with their expectations.

PMI nurses who join the program during the assessment have an interview with SB at which time the details of the trial are explained and the model and content of the HVs of the intervention group are presented.

### Data Collection

The data are collected by SB during the child's fourth and 24th month and HVs and stored at the CPN under conditions guaranteeing confidentiality. Personal data are stored in separate places.

All the details regarding people leaving the trial (lost to follow-up, refusal to continue with the research, child taken into care, etc.) will be recorded for analysis. For the intervention group, data is collected by PMI nurses during HVs, or by SB if the fourth month HV is accessible (family setting, professional situation, daycare arrangements, etc.). For the control group, the same data will be accessible if they have not withdrawn prior to the fourth month visit. If a family leaves the study before the HV at 4 months, SB will attempt to contact this family to obtain this information. If the information cannot be obtained, SB will only have access to the information recorded in the pregnancy notification, which includes the number of dependent children, the mother's age, address, child's date of birth, and PMI follow-up.

For families not wishing to join the research program, the known data, such as address, number of dependent children, and mother's age, will be recorded for analysis to compare consenting families with non-consenting.

If the protocol is not followed, i.e., HVs are not carried out, the dedicated PMI nurse calls the family three times. Should the family not respond, SB phones the family to attempt to understand why parents wish to withdraw from the study. SB may propose adjustments (change of PMI nurse or modification to the HV timetables). If the family maintains its position, the family will be withdrawn from the protocol and their details studied. In addition, the family may still benefit from the usual follow by the PMI nurse if necessary.

During the semi-directed interview at 4 and 24 months, SB establishes whether the families receive similar follow-up (psychologist, psychiatrist, PMI visits, social workers, speech therapist, etc.) in order to analyze these data and link them to the results obtained in assessments.

We have chosen to conduct most assessments at 24 months to meet the primary objective and to limit the number of families lost to follow-up.

#### Scale of Measurement - Tests

The different instruments used are detailed in Table 3 (58–79). We elected to assess the intervention program at 4 and 24 months to use the same scales at both stages of the child's development. If not possible, two different scales are used in the assessments at 4 and 24 months (EPDS-BDI), and they present a good correlation (91). The choice of tests has proven restrictive given the criteria used, namely a validated test in French, and without specific and extended training (92, 93).

#### Socio-Demographic and Family Data

Socio-demographic and family data are collected for both groups: age, level of education, occupation, and family situation, for both parents. For the child: gestational age, gender, birth order within siblings, and birth weight. Information is collected on type of birth, child care arrangements, school attendance, and age on starting school, whether planned or unplanned birth, feeding (breast or bottle), father's involvement in the PERL study, whether or not the family is receiving other support measures, etc. Finally, birth trauma and the presence of any serious psychopathology in the parent will be scored.

During the interview, SB will record how the parent feels about the child, the way the parent sees themselves as a parent, their responses to crying/opposition from the baby, and parent experience of the intervention. SB collects information about how the parents accommodate regular HV from a PMI nurse and how they benefit from this support.

### Data Analysis

All the conditions of use of the tests will be checked before using them. A significant level is <0.05 for all tests.

#### Descriptive Analysis

A descriptive analysis will be performed on the socio-demographic data, considering vulnerability factors, the family's experience of the intervention, the reasons given for refusing to take part in the research program, and the discontinuations.

#### Comparative Analysis

The effects of the support will be assessed by comparison with the control group. The student's *t*-test will be used for the comparative analyses of the quantitative variables. The chi-squared (χ^2^*)* test will be used for the comparative analyses of the qualitative variables. We will also compare the basic characteristics of populations who refused to take part with those who agreed to be included in the study.

#### Correlation Analysis

Spearman's rank correlation coefficient will be used to test the correlations between the scores obtained on the different scales and the impact of the parental representations on the scores obtained with the different scales.

#### Longitudinal Assessment

A longitudinal assessment will be carried out for both groups to measure the evolution of the test scores from the fourth to the 24th month. A Cox model will be used, with the baby's development (under/over average development) as principal judgment criteria.

## Discussion

### Expected Findings

The PERL study proposes and evaluates preventive intervention home support starting at the earliest age to support parent-child interactions and the child's development, which is integrated into the HVs made by PMI nurses. In line with the priorities set out in the national health policy, this action-research project is in keeping with the principle of reducing social inequalities in healthcare access in France.

### Strengths and Limitations

In the PERL study, the intervention is available to the general population, without applying vulnerability criteria as is usually the case (94–97). PMI is a universal service, accessible to the general population. Recruitment is carried out via a universal approach, by making the same system available to the population, irrespective of social status. According to work conducted by the University of British Columbia (34), child vulnerability exists in all socioeconomic layers of our society and the majority of vulnerable children are found in families with middle-class socioeconomic status. The PERL study approach is available to all families who attend the meeting at the recruitment stage where HVs are proposed. No meetings are arranged with families who refuse from the outset.

The first limitation of this system is that families have several opportunities to refuse before they are included in the study. A system of natural selection is therefore in place and families who agree to participate have demonstrated their motivation and desire to receive preventive support regularly. The involvement of families is key in the success of the PERL study (16, 94). It was not possible to meet families during their visits to maternity hospitals given the high number of maternity hospitals involved (5) and their geographical spread (1,576 km^2^), which would have allowed us to limit the opportunity to refuse participation.

The second limitation concerns the constant and necessary adaptation to the difficulties encountered in the field, as reported by the PMI nurses. The PERL study protocol was based on recognized theoretical bases that are transferable into current PMI practices. However, as for all action research, adaptations were necessary due to professional constraints (time allocated to the tool, lack of staff, and adaptation of training, etc.) and comments from PMI nurses regarding the practical implementation of the protocol in the field.

The third limitation concerns a possible contagion effect between the two groups of the study. Most of the PMI nurses in the trial area were taking part in the PERL study and so receive the training and supervision and are involved in working and exchange days. They are therefore all confronted with a potential change to their routine practice. We will therefore identify the families of the control group benefiting from a “traditional” PMI follow-up.

This information is collected during the assessment HVs (at 4 and 24 months). In the case of 525 the PERL study, PMI nurses needed to change their practices. These changes took place on several levels. First, from a structural and organizational point of view, it influences and encourages dedicated time for training, supervision, and analysis of practices. Second, the HVs and the PMI nurses: use more observation for some or more play; are less direct in the exchanges; actively aim to highlight parenting skills; adapt the advice they provide to parental requests through guidance; and do not give identical advice to all parents (prevention recommendations). A third change took place in that, undertaking more HVs than usual allows PMI nurses to respect the parenting process, which is set up at different stages for each parent. Previously, the PMI nurses conducted a single postnatal HV for families that were not classed as vulnerable, mainly primiparous, and when the workload allowed. They would then give all the prevention recommendations during this single HV and make themselves available for follow-ups if needed. The meetings could be very directive and place little focus on the dimension of early mother-child interactions or the mother's thymia. For so-called vulnerable families, the follow-ups could be more “educational”, with objectives that the PMI nurse would set, but not necessarily at the parents' request.

There are further limitations concerning the fact that SB is involved in promoting the study, recruitment, and conducting/processing assessments. To address this, tests will be analyzed blindly by the coordinator and other psychologists working on the protocol for video-based tests (Mini Q-SORT, AQS, early interactions at Bobigny, Brunet Lézine revised). The ADBB test will be analyzed by other trained professionals.

Early referrals may also be made to specialist therapy services (in particular for the intervention group), which may impact the scores in some tests. The purpose of early prevention programs is to direct parents, from the outset, to care services and organizations that can support them with their difficulties (care services, social services, etc.) (27–30, 34).

The final limitation lies in the small sample size of the study. We are well-aware that, given the small sample size, we may not be able to highlight any significant difference on the primary endpoint but will, however, gain quantitative and qualitative indicators of the impact of this device. In addition, if the results on the scales are not significant, a qualitative analysis of the results will be carried out.

### Clinical and Research Implications

Depending on the outcomes, the study aims to propose further development and roll out this HV model nationally. To facilitate this, an analysis of the processes and mechanisms will be carried out in addition to the longitudinal and comparative assessment of the PERL study. This analysis involves defining what effects are produced by the levers involved and how common and non-specific psychological factors (such as professional alliance, time allocated, theoretical backgrounds, etc.) impacted the possible effects of HVs. It will also focus on the mechanisms linking the intervention to its outcomes, description of the implementation and the processes, and will analyze the effects of the context, particularly the social context, on processes and outcomes. This analysis is the subject of a specific protocol, drawn up by the school of public health in Nancy.

It is important to develop primary and early prevention action plans in the perinatal period to investigate factors that promote early child development and to suggest appropriate support models. It is essential that these actions are carried out by PMI nurses as they have specialist knowledge of early childhood and are on the front line of primary prevention.

The PERL study is interesting at a local level, where it could define and structure professional early childhood interventions more efficiently. At the national level, it contributes to the development of public health policies aimed at recognizing, monitoring, and preventing problems in early childhood. It is important to implement this program in a general population as developmental delays and relational difficulties are observed across all socio-economic classes. Moreover, it may pave the way for a more personalized follow-up program for the more vulnerable families, and lead to adjustments in future programs on mental health primary prevention in young children.

It will take several years for the strategic effects, especially those relating to child relations and development, to bear fruit (“sleeper effect”) (14). It would therefore be interesting to implement these measures in the longer term to establish whether the anticipated effects are consistent over time.

Family recruitment was completed in December 2019. The fourth month evaluations were completed in July 2020, and 24-month evaluations are currently underway. The first results will be analyzed in 2021, examining the characteristics of the study population, homogeneity of the groups, and the scores on the tests at 4 months. The comparative analysis will begin in 2022, once the 24-month evaluations are completed for both groups. The final comparative analysis will be carried out in 2024–2025, when all the 4 ERTL tests have been carried out by the PMI nurses.

## Ethics and Dissemination

### Consent, Withdrawal, Information Provided

Consent to take part in the study is obtained in the eighth month of pregnancy, during the study presentation HV. An information sheet detailing information about the research is read out to the family. The sheet is kept with the consent form (signed by the families that agree to take part). This form is signed by both parents if both have parental authority, and failing that, by the parent with exclusive parental authority. Following this, the family is included in the research.

A document relating to the use of images must be also signed by the parents to ensure they consent to the use of the videos required to score the filmed assessments.

The families are informed that they have a right to be updated on the outcomes of the research, publications, and communications concerning the trial.

### Ethical Principles and Safety

This is version six, dated July 4, 2019.

The PERL study was approved by the Lille IV C*omité de Protection des Personnes* (CPP, the Institutional Review Board) on 04/07/2019 (initial approval on November 22, 2017), and the *Agence Nationale de Sécurité du Médicament et des produits de santé* (the National Agency for Medicines and Health Products Safety) has been informed of this trial.

Authorization was granted by the *Commission Nationale de l'Informatique et des Libertés* (the national commission for information technology and civil liberties) on January 30, 2018. The PERL study has been registered on ClinicalTrials.gov under number: NCT 03506971.

For any changes to the protocol, an application will be made to the Lille IV CCP. As soon as the CPP provides agreement, we will inform the families of the change, should this change impact them.

The protocol complies with the ethical principles established by the Declaration of Helsinki (98).

In accordance with the General Data Protection Regulation (99), participants have been informed of their rights to discontinue the trial and to access, rectify, object to or limit the use of the data collected, as well as the nature of the data, the purpose for which it is collected, the length of time it is retained, and the names of the people in charge of this study.

When families are included in the program, the data is systematically anonymized. A number is allocated to each family and no identifying information is linked to the data collected.

This trial does not require any specific vigilance monitoring. Any adverse effects observed or reported by the families will be recorded in the trial report book and SB will inform the relevant authorities if necessary.

The PERL study is supported by a multi-disciplinary scientific committee that meets every 5 months to review the progress of the research program and ensure that all ethical and methodological criteria are being met. A Technical Committee meets every 6 weeks to address specific issues relating to the implementation of the research program on the ground.

## Ethics Statement

The studies involving human participants were reviewed and approved by Comité de Protection des Personnes Nord Ouest IV CPP, 2017 A00896 47. Written informed consent to participate in this study was provided by the participants' legal guardian/next of kin.

## Author Contributions

SB, the coordinator of the research program, has been involved in designing the trial and wrote the article and the protocol. She also generates the randomization, recruits the families, collects and processes statistical data, attends steering committee and technical committee meetings, undertakes supervision, and coordinates the working and exchange days with the pediatric nurses. BK coordinates medical research and has been involved in designing the trial and attends steering committee and technical committee meetings. FL has been involved in designing the methodological aspects of the trial, attends technical committee meetings, undertakes analysis data, and contributed to writing this article. M-CC coordinates the implementation of the trial within the PMI. All authors read and approved the final manuscript after revising it for important intellectual content.

## Conflict of Interest

The authors declare that the research was conducted in the absence of any commercial or financial relationships that could be construed as a potential conflict of interest.

## Abbreviations

HV: Home visit
SB, Sophie Buchheit: Coordinating psychologist
PMI: Protection Maternelle et Infantile (maternal and child welfare services).
